# Supplementary material for: Are carbon emissions trading and green financial instruments synergistic? -Comprehensive quantitative research based on content analysis
Source: PLoS One. 2024 Mar 7;19(3):e0298601. doi: 10.1371/journal.pone.0298601 (PMC10919601; doi:10.1371/journal.pone.0298601)
Supplement: S1 Data — (ZIP) [file pone.0298601.s001.zip › Data/地级市Z_F工作报告.zip!/╔╜╢1⁄2/╠⌐░▓╩╨/└┤╫╘╣1⁄2╓┌║┼í╛┬φ┐╦╩2╛▌═°í┐_2008.docx]

各位代表：

现在，我代表市人民政府向大会作工作报告，请予审议，并请市政协委员和其他列席人员提出意见。

一、过去的五年，全市经济社会发展取得显著成绩

过去的五年，全市人民在中共泰安市委的领导下，以邓小平理论和“三个代表”重要思想为指导，认真贯彻落实科学发展观，坚持市委确定的“一个目标、三大重点、四个坚定不移”的发展思路，解放思想，团结拼搏，求真务实，锐意进取，全市改革开放和现代化建设取得显著成绩。

（一）经济综合实力显著增强。预计2007年全市实现生产总值1210亿元，是2002年的2.14倍，五年年均增长16.4%。其中一二三产业增加值分别达到130亿元、675亿元和405亿元，年均增长5.9%、20.2%和14.9%；完成地方财政收入64.2亿元，是2002年的2.7倍，同口径年均增长25.97%；实现社会消费品零售总额375.5亿元，年均增长15.9%；全市金融机构各项存、贷款余额达到792.3亿元、543.8亿元，年均增长17.7%和16.5%。“十五”计划顺利完成，“十一五”规划进展顺利。

（二）经济结构及运行质量逐步优化提高。三次产业结构由15.6：47.8：36.6调整为10.7：55.8：33.5。工业规模不断壮大，主导产业、骨干企业、知名品牌培植取得明显成效。全市规模以上工业企业达到1297家，完成增加值560亿元、实现主营业务收入1870亿元、实缴税金77亿元，年均分别增长27.2%、40.6%和30.7%。其中主营业务收入过10亿元、实缴税金过5000万元的企业达到23家、17家，分别增加18家和12家。中国名牌产品达到6个，中国驰名商标3个。全市规模以上高新技术产业产值占规模以上工业总产值的比重达到26.8%。旅游服务业发展加快，实现国内旅游收入104.8亿元，年均增长25.9%。

（三）新农村建设取得较大进展。粮食生产保持稳定，农业结构调整力度加大。全市高值田、无公害、绿色、有机农产品基地面积分别达到140万亩、187万亩、180万亩、23.4万亩，分别增加59万亩、95.8万亩、173.5万亩、17.4万亩；奶牛存栏17.7万头，增加15.7万头；森林覆盖率30.9%，提高8.1个百分点；规模以上农产品加工龙头企业达到213家，其中过亿元的27家；农村合作经济组织发展到1428个。农村生产生活条件得到较大改善，行政村通油路率达到96%，自来水普及率达到95%，完成了50户以上自然村“村村通”广播电视，累计建成农村沼气池7万个，完成新一轮改厕15万户，农田水利基本建设、土地开发整理、农业机械化等都取得新成绩。重大动植物疫病防控得到加强。农村税费改革顺利完成，全部取消农业税和农业特产税；认真落实各项支农惠农政策，累计发放粮食直补、良种补贴、农机补贴、农资综合直补和库区移民扶持等资金共计9.3亿元；农村财务委托代理工作被中宣部作为全国十大典型进行推广。

（四）大项目建设取得大的突破。全社会累计完成固定资产投资2245.6亿元，年均增长27.1%。实施建设投资亿元以上的工业、商贸、旅游项目383个，投资总规模949.5亿元，投资43.3亿元的泰山抽水蓄能电站一期、27.9亿元的国电石横电厂三期扩建、20亿元的泰山盐化工一期、12亿元的光彩大市场一期等一批单体投资过10亿元的项目建成投产运营；投资20亿元的宝龙城市广场、20亿元的岱银天梭工业园、23亿元的山东众泰发电、15亿元的石横国际示范园、53.6亿元的华兴纺织工业园、15亿元的兆宇石油管制造等十几个投资过10亿元的项目建设进展顺利。项目建设的有力推进，促进了经济实力的增强，提升了产业结构档次，增强了企业的竞争能力和发展后劲。

（五）改革开放取得重大进展。市县（市、区）企业改制基本完成，国有资产管理体制改革逐步深化，市直行政事业单位国有资产管理体制改革全面推进。行政审批制度改革、高新区管理体制改革、泰山景区管理体制改革、城市综合执法体制改革、城区警务体制改革、粮食购销企业改革等有效实施。事业单位改革、财税金融体制改革、社会保障等项改革也都取得新进展。对外开放水平明显提高，对外交流合作日趋活跃。全市总投资3000万元以上的国内招商引资项目到位资金492亿元；累计新批利用外资项目484个，实际利用外资27.6亿美元，年均增长11.6%和69.5%；完成进出口总值40.5亿美元，年均增长33.9%，其中出口28.3亿美元，年均增长29.6%。

（六）城镇面貌发生明显变化。修编完成了泰城及各县市新一轮总体规划。加大城市基础设施和环境综合整治力度，泰城先后实施完成了环山路、龙潭路南段、泮河大街、东湖和南湖公园、天平湖公园、第二污水处理厂、垃圾处理场等一批城市基础设施项目，共改造、扩建和整治城区道路81条，面积700万平方米，新建改造游园公园7个，新增公共绿地面积327万平方米，人均绿地面积达到14.6平方米，日处理污水能力16万立方米。县市驻地和小城镇规划建设与管理取得新成绩。泰安被评为“国家历史文化名城”，再次荣获“国家卫生城市”称号；新泰市被评为“国家园林城市”和“中国优秀旅游城市”。全市城市化水平达到47%。高新区基础设施建设、管理体制创新、项目建设、社区建设等取得显著成绩，载体功能和辐射带动作用进一步增强。

（七）“和谐泰安”建设取得初步成效。市区居民人均可支配收入、农民人均纯收入分别达到13700元、5300元，年均增长13.2%和11.1%，城乡居民生活水平明显提高，享有的公共服务不断增多。创新型泰安建设全面启动，应用技术研究与开发经费累计投入6195万元，获得省、国家科技进步奖235项和6项，省以上高新技术企业发展到174家，输变电设备和非金属新材料两个产业集群被认定为国家火炬计划特色产业基地；泰安被列为全国知识产权试点市。基础教育得到加强，“两基”成果得到巩固提高，“以县为主”的农村义务教育管理体制不断完善，教育经费保障机制初步建立，“两免一补”政策全面落实，投入3.2亿元新建农村中小学校舍62.4万平方米，普通高中、职业教育、高等教育都有新的发展。文化事业繁荣发展，文化信息资源共享工程扎实推进,泰山石敢当习俗列入国家非物质文化遗产名录，有9件作品获省以上精品工程奖。城乡预防保健、疾病控制、医疗救治、农村三级卫生服务网络、城市卫生服务体系建设得到加强，新型农村合作医疗农民参合率达到96.7%。就业再就业稳定增长，全市累计新增城镇就业再就业38.7万人，农村劳动力累计就地转移和在外务工人员均超过60万人。社会保障制度和体系逐步健全，各项社会保险费收支总规模达到167.7亿元，城乡低保、五保供养、救灾救济等制度逐步完善，城镇居民基本医疗保险开始启动，被列为国家级试点城市。制定出台了经济适用住房管理暂行办法，实施了4.7万平方米的经济适用住房建设，市和各县市区全部建立了廉租房租金补贴制度；全市累计归集住房公积金23亿元，发放贷款15.6亿元。爱国卫生运动深入推进。全民健身活动广泛开展，竞技体育取得优异成绩。人口和计划生育工作连续10次获省考核先进单位称号，低生育水平保持稳定。耕地保护、矿产资源开发秩序和河砂资源综合整治得到加强。泰山荣获“世界地质公园”称号，被评为十佳“全国文明风景旅游区” 和全国首批5A级旅游景区。扎实推进创建国家环保模范城市和生态市建设工作，狠抓污染物减排，环境保护工作取得明显成效。“三城联创”等精神文明创建活动深入开展，民主法制建设不断加强。双拥共建、民兵预备役建设、人民防空工作取得新成绩，泰安第四次荣获“全国双拥模范城”称号。“平安泰安”建设卓有成效，广播电视、新闻出版、外事侨务、民族宗教、气象、地震、档案、史志、老龄、残疾人等项社会事业全面进步，社会保持和谐稳定。

（八）政府施政能力不断提高。深入贯彻《公务员法》、《监督法》、《行政监察法》和《全面推进依法行政实施纲要》等法律法规，健全完善了执法责任追究、执法评议考核、行政效能监察和举报投诉等制度。加强行政审批中心建设，建立了网络审批服务平台，对行政许可和非行政许可项目进行了全面清理规范。加快推进政务信息化步伐，大力推行政务公开，开通了泰安政府网站、便民服务电话、民生直通车网站，被评为全国政务公开先进单位。加强政府廉政建设，大力推进市县机构改革，加快推进政府职能转变，建立完善了决策目标、执行责任、考核监督三个体系，政府服务效率和质量有了明显提高。诚信建设深入推进，“投资泰安、稳如泰山”的品牌效应逐步显现。

各位代表，过去五年，是全市改革开放和全面建设小康社会取得重大进展的五年，是经济综合实力显著增强的五年，是城乡面貌发生显著变化的五年，是人民群众得实惠最多和生活质量大幅提升的五年。这些成就的取得，是在历届政府打下的良好基础上，在市委的坚强领导下，全市各族人民团结奋斗，开拓进取，顽强拼搏，真抓实干的结果；也是驻泰各单位和各界人士全力支持、自觉融入泰安经济与社会发展的结果。在此，我代表泰安市人民政府，向全市广大干部群众、各民主党派、工商联、无党派爱国人士，向人民解放军和武警驻泰部队及驻泰单位，向关心支持泰安发展的港澳台同胞、海外侨胞及海内外朋友，表示崇高的敬意和衷心的感谢！

在充分肯定成绩的同时，我们也清醒地看到发展中还存在不少困难和问题，政府工作也有许多不足。一是思想解放程度不够，危机忧患意识、发展欲望、市场竞争观念需要进一步增强。二是经济综合实力还不够强，结构性矛盾依然比较突出，企业自主创新能力不强，节能降耗、环境保护压力比较大，转变发展方式的任务繁重。三是改革开放的力度需进一步加大，体制性、机制性障碍依然存在，利用外资、进出口总值基数低、绝对额小。四是和谐泰安建设还存在一些薄弱环节，农民持续增收的基础还不牢固，社会保障覆盖面和保障能力仍需进一步提高，涉及人民群众切实利益的问题还比较突出，社会还存在一些不稳定因素。五是发展环境需要进一步优化，行政效能和服务质量需要进一步提高等等。对此，我们必须高度重视，以更加高昂的斗志、更加有力的措施，强力推进全市经济与社会又好又快发展。

二、统筹谋划，努力实现今后五年科学发展的新跨越

今后五年是立足新的起点，实现科学发展，全面建设小康社会的重要时期。政府工作总的要求是：认真贯彻党的十七大精神，高举中国特色社会主义伟大旗帜，坚持以邓小平理论和“三个代表”重要思想为指导，以科学发展观总揽全局，按照市第九次党代会确定的建设“富裕文明和谐”泰安的目标要求，紧紧围绕转变经济发展方式和完善社会主义市场经济体制，突出体制机制创新，突出经济结构调整优化，突出自主创新和节能减排，突出解决民生问题，突出统筹协调和谐发展，大力推进文化建设、社会建设和民主法制建设，调动发挥各个方面的积极性和创造性，努力实现经济与社会又好又快发展。政府工作的奋斗目标是：

——经济综合实力明显增强。全市生产总值年均增长13%以上，到2012年力争再翻一番；地方财政收入年均增长15%；社会消费品零售总额年均增长15%以上。

——经济结构进一步优化升级。现代农业快速发展，产业化水平进一步提高；现代工业体系逐步形成，先进制造业、高新技术产业快速发展；旅游支柱产业地位巩固提升，现代服务业繁荣发展，三产占经济总量的比重有大的增长。

——改革开放的动力与活力日趋增强。各项改革尤其是重点领域的改革进一步深化，科学发展的体制机制基础进一步增强。实际利用外资年均增长15%以上；外贸进出口年均增长15%以上。

——发展方式得到显著转变。自主创新能力明显增强，高新技术产业产值占规模以上工业总产值的比重年均增长3个百分点。循环经济和节能减排实现较大突破,万元GDP能耗、取水量降低20%和24%以上。生态环境质量明显改善，生态市建设取得显著成效。

——城乡面貌发生大的变化。新农村建设取得显著成效，城乡一体化进程进一步加快；泰城中心城市与县市驻地城市的品位、功能明显提升完善，要素承载能力进一步增强，人居环境更加和谐优美。

——人民生活水平进一步提高。市区居民人均可支配收入和农民人均纯收入年均增长8%和7%。居民消费结构日趋优化，享受的社会公共服务显著增多。

——社会更加和谐稳定。人口自然增长率控制在7.1‰以内。就业再就业平均每年增加5万人，城镇登记失业率控制在3.5%以内。社会保障体系更加健全完善，保障能力进一步增强。社会文明程度明显提升，民主法制建设得到加强，人民权益得到有效保障，社会公平正义得到切实维护，社会管理和公共服务水平不断提高，“平安泰安”建设更加深入。

十七大开启了全面建设小康社会的新征程。地区间新一轮又好又快发展的竞争已经展开，机遇与挑战并存。机不可失，时不我待。紧紧抓住机遇，科学谋划未来，创造新的发展优势，实现新的发展，是广大人民群众的期盼，更是政府工作的神圣职责。在工作的指导和把握上，要做到“五个必须”：必须始终坚持解放思想，增强责任意识，创新观念，拼搏进取，大胆探索，勇于实践；必须始终坚持以科学发展观统揽全局，紧紧扭住发展第一要务不放松，聚精会神搞建设，一心一意谋发展，创新发展理念，转变发展方式，提升发展质量，做到稳中求进，好字优先，又好又快；必须始终坚持统筹兼顾、重点突破，紧紧抓住牵动全局的重点工作、事关群众利益的突出问题，强力推进，力求实现大的突破，整体推进城乡、经济与社会、改革与开放、人与自然协调和谐发展；必须始终坚持市委确定的战略目标不动摇，以大项目建设为基本抓手，以自主创新、节能减排、招商引资和体制机制创新为切入点，强化工作措施，加大工作力度，努力实现又好又快发展；必须始终坚持以人为本，更加重视改善民生，实现好、维护好、发展好人民群众的根本利益，做到发展为了人民，发展依靠人民，发展成果由人民共享，凝聚全社会的力量，形成加快科学发展的强大合力。

三、立足转变，强力推进2008年全市经济与社会又好又快发展

2008年，是全面贯彻落实党的十七大精神的第一年。按照市委的总体部署，与“十一五”规划相衔接，全市经济社会发展的主要预期目标和约束性指标为：生产总值增长14%；地方财政收入同口径增长16%；万元GDP能耗、取水量降低4.7%和7%；社会消费品零售总额增长18%；全社会固定资产投资增长18%；外贸进出口增长18%，实际利用外资增长15%；市区居民人均可支配收入和农民人均纯收入均增长8%；城镇登记失业率控制在3.5%以内，人口自然增长率控制在5.8‰以内。在实际工作中力求发展得更好一些、更快一些。

（一）扎实推进社会主义新农村建设。坚持城乡统筹，走中国特色农业现代化道路，建立以工促农、以城带乡长效机制，繁荣发展农村经济。一是加大农业结构调整力度。在确保粮食安全的前提下，积极推进“一村一品百村示范千村推进工程”，大力发展高产、优质、高效、生态、安全农业和优势特色产品，突出设施农业、有机蔬菜、苗木花卉基地建设和以“奶业富民工程”、生猪生产为主的畜牧业，尽快把优势产业做强、把特色产业做精；鼓励扶持发展壮大农业龙头企业，建立完善农村市场体系，大力发展农村合作经济组织，加快农业标准化生产，强化农产品质量安全和动植物疫病防治，加大农业科技培训和推广力度，加快推进传统农业向现代农业转变。二是加强农村基础设施建设。加大公共财政对农村的支持力度，建立以政府投入为引导，银行信贷、社会资本广泛参与的多元化农业投入机制。重点抓好各类水库除险加固、水土流失综合治理、节水灌溉、植树造林和农业综合开发、基本农田建设，进一步提高农业机械化装备水平，增强农业综合生产能力。继续搞好农村“路水电医学”、广播电视村村通和养老院、文化站等基础设施和公共事业建设，大力发展沼气、太阳能等清洁能源，深化新一轮农村改厕，加大村镇环境综合整治力度。三是多渠道增加农民收入。大力发展农村二三产业，实施好农村劳动力转移培训阳光工程，拓宽农民增收渠道，全市新增农村劳动力转移就业5.8万人。同时，认真落实各项支农惠农政策，加强对农民负担的监管，防止农民负担反弹。

（二）做强做大工业经济。按照走新型工业化道路的要求，大力推进信息化与工业化的融合，逐步形成主导产业突出、传统产业增强、骨干企业众多、创新能力较强的工业体系。以结构调整为主线，以骨干企业和优势产品为依托，大力发展先进制造业，努力提高先进制造业在工业经济中的比重，带动和促进工业经济整体素质和竞争力的提升。围绕“三个一批”，加快培植壮大先进制造业主导产业，突出抓好输变电设备、汽车及零部件、无机非金属新材料、精细化工等产业链条的延伸、拓展，培植形成一批规模大、效益好、市场竞争力强的先进制造业骨干企业和企业群体。高度重视高新技术产业发展，努力引进和培植壮大一批拥有自主知识产权、具有较强国际竞争力的高新技术企业，大力发展电子信息、新材料、光机电一体化、新能源和高效节能高新技术产业，年内高新技术产业产值占规模以上工业总产值的比重达到29.8%。加大传统产业改造力度，加快利用现代企业管理理念和先进适用技术提升企业技术装备水平和整体素质，全年计划完成技术改造投资370亿元。

（三）繁荣发展旅游服务业。紧紧围绕“打造旅游目的地、创建国际旅游名城、建设旅游经济强市”目标，进一步做好“吸引人、留住人”的文章，努力提升旅游业的档次和水平。理顺创新体制机制，统筹整合旅游资源，以项目建设为载体，下大气力抓好泰山景区一个龙头，泰城城区休闲、旅游度假区、泰山东麓、徂徕山景区、莲花山景区、东平水浒文化景区等八大板块的开发与提升，真正形成大泰山、大旅游的发展格局，增强旅游竞争能力。重视抓好旅游市场主体培育，引进、培强、做大一批旅游骨干企业。高度重视旅游品牌建设，努力开发一批精品或特色旅游产品，打造一流的旅游服务环境。坚持政府引导、市场运作、企业经营、社会参与原则，创新理念，拓宽思路，落实鼓励扶持政策，优化发展布局，突出发展特色，强力推进现代服务业快速发展。大力发展现代物流、金融保险、科技信息、商务服务等现代服务业，着力实施好“1030”工程，加快培育10个市级服务业集聚区、30个服务业重点企业，集中力量抓好南京顺天数码城、泰山物流中心、红星美凯龙家具城、新时代装饰材料广场等项目建设；规划建设一批特色商业街、小吃街和大型购物设施，大力发展文化娱乐、康体休闲、卫生保健等生活性服务业；加快服务项目、产品、方式创新，积极扩大企业、公共事业机构和政府服务外包业务，积极承接国际服务业外包，引进、培育一批服务业知名品牌，促进服务业上档次、上水平。积极推进“万村千乡市场工程”建设，加快建设农村现代商品流通网络。千方百计增加城乡居民收入，培育消费热点，优化消费环境，促进消费增长，增强消费对经济的拉动作用。

（四）强力推进大项目建设。项目建设特别是大项目建设，是增强后劲、培植财源、扩大就业、实现科学发展的基本抓手和有效载体，是一个地区经济社会又好又快发展的必由之路和重要支撑。一是集中力量抓好一批续建和新开工大项目。突出抓好玻纤两条8万吨池窑拉丝生产线、泰开工业园二期、泰山啤酒40万吨扩建、泰山盐化工二期、煤化工基地、汇源PET项目、华阳化工园区、瑞星化工水煤浆汽化、华强泰山文化旅游产业园、中信旅游等100个投资过亿元的工业、商贸、旅游大项目。二是搞好项目的策划储备。按照“投产一批、开工一批、储备一批”的要求，紧紧围绕结构调整、自主创新和节能环保、提升城市综合竞争力、培植财源，依托高层次专家咨询机构，论证、策划、储备一批符合国家产业政策、科技含量高、市场前景好、无污染，能持续提供税源的大项目、好项目，重点抓好一批百亿扩规项目的策划和实施建设。三是创新完善大项目建设推进机制。强化重大项目领导包保责任制，建立完善项目建设、利益分配、督查考核与合力推进机制，对列入市重点的项目，实行集中调度和专项督查制度。在国家实施稳健财政政策和从紧货币政策的形势下，多动脑筋，多想办法，千方百计破解资金、土地等难题，保项目落地，保项目建设。加大政策引导力度，努力推动骨干企业上市。对一些重大项目，坚持特事特办、急事急办，尽全力做好争取和服务工作。四是加强企业家队伍建设。创新完善企业家培训、激励机制，努力营造一个尊重企业家、有利于企业家成长的良好氛围，让众多企业家的创新创造智慧竞相迸发，让优秀的企业家大量涌现。

（五）加大自主创新和节能减排力度。紧紧围绕建设创新型泰安，坚持创新驱动，做好“自主知识产权、消化吸收再创新、科技成果转化”三篇文章。进一步深化改革，加大投入，依托企业技术中心、工程技术研究中心、科技孵化中心以及各类科研机构，突出重点领域和关键技术，整合各类科技资源，加大创新力度，增强创新能力。重点培植20家自主创新能力强、产品市场前景好的创新型企业，20家各级认定的企业技术中心，专利申请量和授权量分别达到2100件、800件。加快建立以企业为主体、市场为导向、产学研相结合的技术创新体系，引导和支持创新要素向企业集聚，促进科技成果向现实生产力转化。大力实施“人才强市”战略，创新人才培养、流动、激励机制，继续推进“岱下英才”等人才培养工程，在重点产业和重点领域造就一批高层次研究型、复合型人才；营造鼓励创新的体制机制和环境，在全社会形成尊重知识、尊重人才、尊重劳动、尊重创新、尊重创造的浓厚氛围。

坚持节约资源和保护环境的基本国策，严格落实目标责任制，加大依法治理力度，强化企业社会责任，打好节能减排攻坚战。大力推广十大类节约技术，突出抓好重点领域、重点行业、重点企业的节能减排，加大对冶金、电力、建材、化工等重点企业的技术改造力度，坚决淘汰落后的生产能力；积极开发利用太阳能、生物质能等清洁能源和可再生能源。集约利用土地，严格保护耕地，加强矿产资源、河砂资源的管理和有序开发。保护和节约水资源，建设节水型社会。大力发展循环经济，努力构筑企业、行业、社会三个层面的循环经济体系，重点推进实施“1623培育计划”。大力实施《泰安生态市建设总体规划》，加快推进南水北调、碧水行动、生态市等项目建设，重点加强水、大气、土壤等污染防治，改善城乡人居环境，确保生态环境安全。

（六）进一步深化改革开放。毫不动摇地坚持改革方向，着力构建充满活力、富有效率、更加开放、有利于科学发展的体制机制。深化国有企业改革，加强国有企业股权管理和国有资本运营，健全现代企业制度，增强企业发展活力。健全完善各类国有资产管理体制和制度，深化行政事业单位国有资产管理体制改革。积极推进投融资体制改革，探索产权融资、项目融资、银企合作、上市融资等多元融资形式。培育壮大劳动力、土地、技术、资本等要素市场，规范发展市场中介组织，加快建设统一开放、竞争有序的现代市场体系。深化财税制度改革，完善政府收支、部门预算、国库集中支付、政府采购、转移支付等制度，强化财政公共服务职能。加强信用和担保体系建设，优化金融生态环境。坚持改制与招商重组相结合，加快市直经营开发服务类事业单位转企改制步伐。加快推进统计制度方法改革，完善统计监测评价体系。深化农村综合改革，积极推进农村水利设施、林权制度改革，稳妥推进农村土地承包经营权自愿合理流转。

坚持“市外就是外”和内外联动、互利共赢，积极融入国际、区域间竞争与合作，在更广领域、更高层次上提升对外开放水平。一是千方百计扩大招商引资。潜心研究国际国内产业转移的规律，充分利用产业、资源、环境、服务等要素，全力做好承接转移和加快发展的文章。突出发展先进制造业、旅游业、现代服务业和现代农业龙头企业，充分发挥企业招商的主体作用和园区、项目的载体作用，创新利用外资方式，加大对国内外大客商、大企业、大集团特别是加大对“央字号”大企业、世界500强、国内500强的招商力度，努力扩大招商引资规模。坚持招商引资与改制重组、技术改造相结合，创新项目引进和建设模式，进一步加大蹲点招商、小团队招商和专业队伍招商的力度，增强招商引资的灵活性和实效性，努力提高招商引资的质量和效益。二是努力扩大对外区域合作与交流。按照省委提出的“一体两翼”战略部署，积极融入省会城市经济圈、参与半岛制造业基地的分工与合作，加强与环渤海经济圈、淮海经济协作区、滨海新区经济技术的联系融合。积极实施“走出去”战略，鼓励引导有实力、有信誉的企业到国外投资办厂、承揽工程与开展劳务合作。三是努力扩大外贸出口。加快转变外贸增长方式，优化调整出口商品结构，扩大具有自主品牌和高附加值产品出口，增加服务产品和农产品出口，大力培植出口骨干企业和利税大户。

（七）高度重视以改善民生为重点的社会建设。认真实施“扩大就业的发展战略”，以落实《就业促进法》为契机，强化政府促进就业的公共服务职能，坚持实施积极的就业政策，完善市场就业机制，统筹城乡，鼓励创业，以创业带动就业。着力解决好“4050”人员、“双零”家庭、被征地农民等困难群体的就业再就业问题，重视抓好高校毕业生就业工作。年内实现城镇新增就业再就业6万人。完善收入分配制度，建立企业职工工资正常增长机制和支付保障机制，着力提高低收入者收入。坚持“保发、促缴、扩面、严管”，进一步健全完善以养老、医疗、失业保险为重点的社会保障体系，扩大社会保险覆盖面，提高社会保障能力；全面推行城镇居民基本医疗保险制度，用两年时间将财政对参加新型农村合作医疗农民的补助提高到80元；积极推进农民工、被征地农民社会保险工作；扩大廉租住房保障范围，加快经济适用房和廉租房建设；规范提升城乡低保工作，加大对特困户、灾民等困难群体的救助，大力发展以扶老、助残、救孤、济困为重点的社会福利事业和慈善事业；加强社会保障资金、住房公积金管理，确保资金安全有效使用。进一步加大“平安泰安”建设力度，健全完善社会治安防控体系和社会矛盾纠纷排查调处机制，深入开展社会治安综合治理和严打专项行动，确保社会稳定；深刻汲取教训，强化安全生产责任落实与监督管理，突出抓好矿山、交通、危险化学品和食品、药品等重点领域的安全生产，坚决防止重特大安全事故；高度重视做好人民来信来访工作，切实解决好涉及群众切身利益的问题，及时化解各种社会矛盾。

坚持教育优先发展战略，以农村义务教育经费保障机制改革和教育均衡发展为重点，巩固提高义务教育，加快普及高中段教育，大力发展职业教育，进一步提高教育教学质量和办学水平。继续大力支持驻泰高校的改革与发展。加快建设覆盖城乡居民的公共卫生服务体系、多层次的医疗保障体系和疾病预防控制、突发公共卫生事件救治、卫生监督执法体系，提高医疗卫生保障和服务水平。大力开展爱国卫生运动，巩固提高“国家卫生城市”成果。发展繁荣文化事业和文化产业，高度重视泰山文化研究，打造泰山文化品牌。坚持把社会主义核心价值体系融入精神文明建设全过程，大力倡导社会主义荣辱观，组织实施社会公德、职业道德、家庭美德、个人品德“四德工程”和“乡村阅读工程”、“农家书屋”建设，不断提高全体公民的思想道德素质。加强体育基础设施建设，深入开展全民健身活动，提高竞技体育水平。依法加强宗教事务管理，认真落实少数民族政策，大力发展少数民族经济。继续加强人口和计划生育工作，稳定低生育水平，提高出生人口素质。进一步完善基层民主管理制度，保障基层群众的民主权利。完善国防动员体制机制，注重民兵预备役质量建设。深入开展双拥共建，巩固发展军政军民团结。加快发展繁荣其他各项社会事业，促进社会全面进步。

（八）更加重视提升和优化发展环境。一是加大软环境建设力度，努力提高行政效率和服务质量。重点抓好行政审批、便民服务电话受理等服务中心的健全完善，加快政务服务信息化，简化“一站式”服务程序，为客商和市民提供全覆盖、零缺陷、高效率的服务环境。加强“诚信泰安”、“文明泰安”建设，进一步彰显“投资泰安、稳如泰山”的品牌效应。二是加大硬环境建设力度。围绕建设“蓝天碧水青山”绿色家园目标，坚持“高起点、严管理、广覆盖”，创新城市发展理念，强化城市规划的龙头刚性作用，保护提升老城区，加快新城区开发建设步伐。重点抓好时代发展线和泰山博物馆、104国道改线、京沪高铁车站组团、城区道路改造、污水治理、市政公用设施等项目建设，积极推进城中村改造和旧小区整治，努力提升泰城的综合服务功能，改善人居创业环境。加大县市驻地城市和小城镇规划建设力度，积极推进城市规划、基础设施建设、管理向城乡结合部和小城镇延伸，提升城镇的公共服务功能和对各类要素的承载能力，让更多的城乡居民切实享受到城乡一体化带来的实惠。按照“一点两区”定位，突出招商引资和项目建设，突出理念机制创新与发展方式转变，进一步加大高新区的开发建设力度，努力把高新区建设成为科学发展、要素集聚、充满活力的现代化新区。坚持高起点、高标准，与城镇规划相衔接，搞好其他各类园区功能配置、产业布局、基础设施的规划建设和机制创新，增强发展的动力与活力。

四、加强政府自身建设，努力提高驾驭科学发展的能力

党的十七大对政府工作提出了新的更高要求。各级政府要按照科学执政、民主执政、依法执政的要求，加快推进政府职能转变，努力提高执政能力和执政水平。

（一）建设学习型政府。政府领导成员及工作人员，要善于学习、勤奋学习、终身学习，认真学习发展了的马克思列宁主义、毛泽东思想、邓小平理论和“三个代表”重要思想，尤其要加强对十七大精神的学习，坚持科学发展观，用最新的理论成果武装头脑、指导实践。加强对市场经济、现代科技、行政管理、法律法规等知识的学习，不断掌握新知识，积累新经验，增长新本领。坚持理论联系实际，学以致用，以学促用，不断提高领导科学发展的能力和水平，取得领导科学发展的实际效果。

（二）建设创新型政府。社会创新首先是政府创新。一是创新理念。牢固树立执政为民、科学发展、统筹发展、和谐可持续发展的理念，以新的理念催生新的举措，以新的举措促进新的发展。二是创新行政管理体制。充分发挥市场配置资源的基础性作用，坚持政企分开、政资分开、政事分开、政府与市场中介组织分开，加快政府职能转变，形成权责统一、分工合理、决策科学、执行顺畅、监督有力的行政管理体制。三是创新工作思路和工作方式。准确把握宏观局势，紧紧抓住机遇，顺势而为，乘势而上；善于用战略系统的思维、开放的视野筹划工作、谋划未来，善于运用市场化和行政推动相结合的办法强力推进工作，善于用改革的思路解决发展中的问题。四是开创工作新局面。进一步增强紧迫感和使命感，解放思想，振奋精神，以时不我待、抢抓机遇的责任意识，以敢于争先、勇于赶超的拼搏精神，以不达目的誓不罢休的工作韧劲，不断开创工作新局面、实现新突破。

（三）建设服务型政府。为人民服务、为经济社会发展服务是政府的神圣职责。牢固树立全心全意为人民服务的意识，正确对待和行使人民赋予的权利，体察民情民意，为群众办实事、解难题、谋实利，做到权为民所用、情为民所系、利为民所谋。按照胡锦涛总书记八个方面作风的要求，牢固树立正确的政绩观，大力加强作风建设，大兴求真务实之风，坚决防止和克服官僚主义、形式主义和弄虚作假行为。强化责任、质量和效率意识，进一步健全完善决策目标、执行责任、考核监督三个体系和快捷高效的工作推进落实机制，把更多的时间和精力放在深入调研、潜心研究工作和抓好督促落实上，尤其加大对重点工程、重大事项、重要工作的调度督查和考核奖惩力度，确保各项工作落到实处，抓出大的成效，让群众得到更加明显的实惠。

（四）建设法治型政府。坚持依法执政，是实现科学发展的重要保证。进一步强化法制意识，认真贯彻落实各项法律法规，加强行政执法队伍建设，提高依法施政水平，努力做到行政合法、程序正当、高效便民、诚实守信。进一步健全完善执法责任、错案追究、效能监察、举报投诉等制度，确保权力依法公正运行，确保权力始终受到监督。坚持向人大报告工作和向政协通报工作制度，认真执行人大及其常委会的决议、决定，自觉接受人大的法律监督和政协的民主监督，认真办理人大代表议案、建议和政协提案。加强同各民主党派、工商联、人民团体和社会各界人士的联系。深入开展“五五”普法和“四五”依法治市，提高全社会自觉学法守法用法的意识，夯实社会和谐的法治基础。

（五）建设阳光型政府。让政府权力在阳光下运行，才能获得人民群众的信赖和拥护。坚持用制度管权、管事、管人，建立健全决策、执行、监督、奖惩等制度规则，严格按照法定权限和程序行使权力、履行职责。加快推进政务公开和公共事业单位办事公开，进一步健全完善政府决策、专家咨询、民主听证、社会公示等各类公开办事制度，拓展公开形式，畅通公开渠道，努力提高政府工作的透明度和公信力。强化审计职能，完善监督机制，加强对领导干部特别是主要领导干部、关键岗位人员人财物管理使用的审计与监督。坚持抓源治本，从严治政，严格落实廉政建设责任制，牢记“两个务必”，认真遵守党的纪律和廉洁自律的各项规定，清廉做人，公正办事，以廉洁勤政的良好形象，赢得广大人民群众的信赖和支持。

各位代表，抚今追昔，我们对泰安的进步感到自豪；展望未来，我们对泰安的发展充满信心。让我们高举中国特色社会主义伟大旗帜，更加紧密地团结在以胡锦涛同志为总书记的党中央周围，在市委的坚强领导下，深入贯彻落实科学发展观，进一步解放思想，开拓进取，埋头苦干，为加快推进“富裕文明和谐”泰安建设进程、全面建设小康社会而努力奋斗！
